# Supplementary material for: Molecular basis of RNA-binding and autoregulation by the cancer-associated splicing factor RBM39
Source: Nat Commun. 2023 Sep 4;14:5366. doi: 10.1038/s41467-023-40254-5 (PMC10477243; doi:10.1038/s41467-023-40254-5)
Supplement: Supplementary file 1 — Supplementary Information [file 41467_2023_40254_MOESM1_ESM.pdf]

Supplementary information

Molecular basis of RNA-binding and autoregulation by the cancer-associated splicing factor RBM39

Sébastien Campagne[1,2,*], Daniel Jutzi[3], Florian Malard[1,2], Maja Matoga[1], Ksenija Romane[1], Miki Feldmuller[1], Martino Colombo[4,5], Marc-David Ruepp[3,*] and Frédéric H-T. Allain[1,*]

[1]ETH Zurich, Department of Biology, Institute of Biochemistry, 8093 Zurich, Switzerland; [2]University of Bordeaux, Inserm U1212, CNRS UMR5320, ARNA Laboratory, 33077 Bordeaux, France; [3]United Kingdom Dementia Research Institute Centre, Institute of Psychiatry, Psychology and Neuroscience, King's College London, Maurice Wohl Clinical Neuroscience Institute, London SE5 9NU, United Kingdom; [4]University of Bern, Department of Chemistry and Biochemistry, 3012 Bern, Switzerland; [5]Celgene Institute of Translational Research in Europe (CITRE), Bristol Myers Squibb, 41092 Seville, Spain.

SC and DJ contributed equally to this work.

*Correspondence should be addressed to allain@bc.biol.ethz.ch, marc-david.ruepp@kcl.ac.uk or sebastien.campagne@inserm.fr

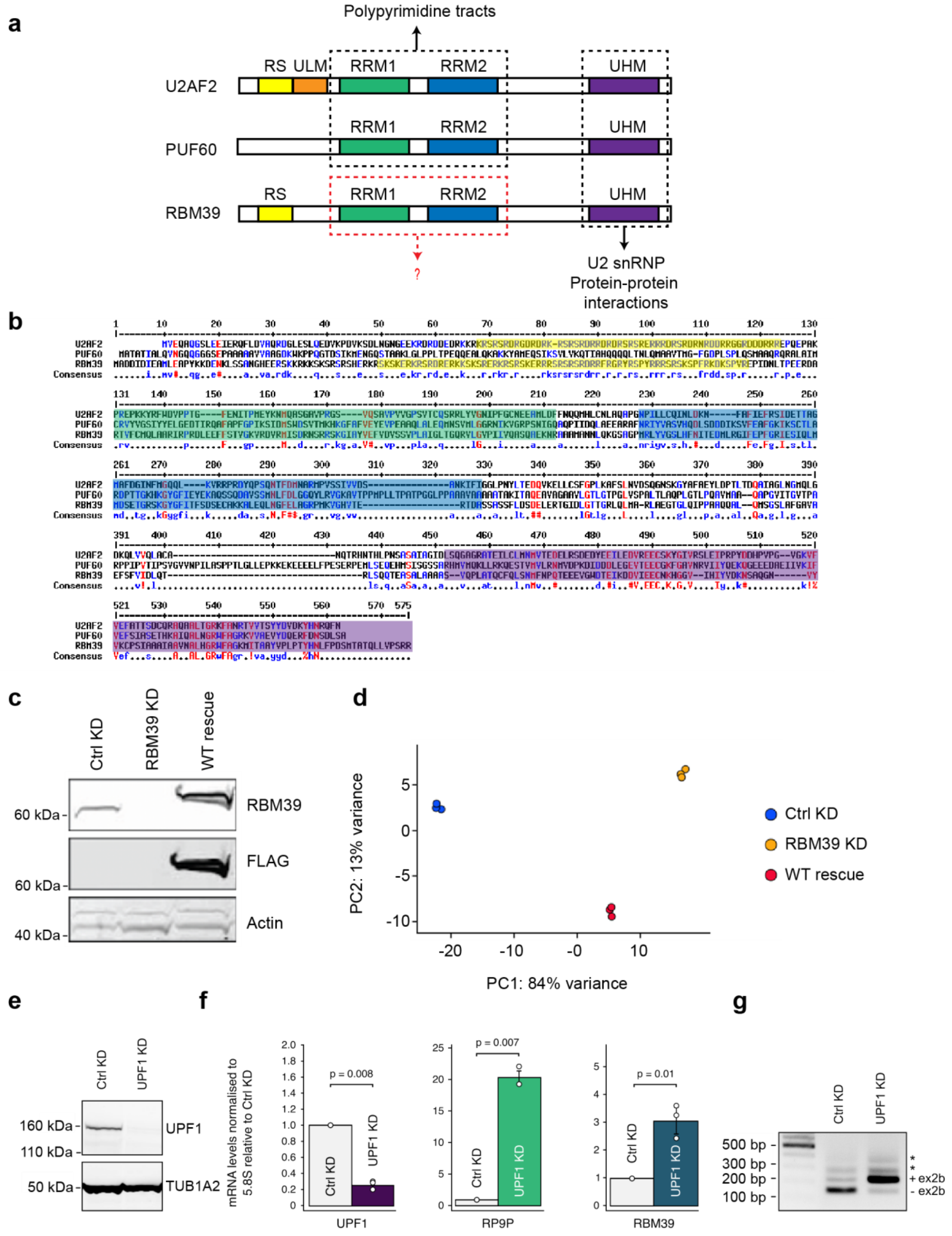

Supplementary Fig. 1: RBM39 is homologous to U2AF2 and PUF60 and it promotes the inclusion of a poison exon in own pre-mRNA. a, Domain organisation of RBM39, U2AF2 and PUF60. **b,** Sequence alignment of RBM39, U2AF2 and PUF60. **c,** Western blot analysis showing the efficiency of RBM39 siRNA knock down and its rescue with FLAG-RBM39. This experiment was repeated three times and led to similar results (n = 3). **d,** Principal component analysis of the 9 samples that were analysed by RNA-Seq. **e,** Western blot showing the knock down of UPF1. **f,** Bar plots showing the

mRNA abundance of UPF1, PRP9 and RBM39 in wild type conditions and after UPF1 knock down. Average values and standard deviations of three biological replicates (n = 3) are shown. P values were computed from log-transformed ratios using two-sided t-test. **g,** Agarose gel showing the accumulation of the RBM39 non-productive mRNA isoform in the UPF1 knock down conditions (UPF1 KD). In control conditions (Ctrl KD), the RBM39 non-productive mRNA isoform is degraded via non-mediated decay pathway. This experiment was repeated three times and led to similar results.

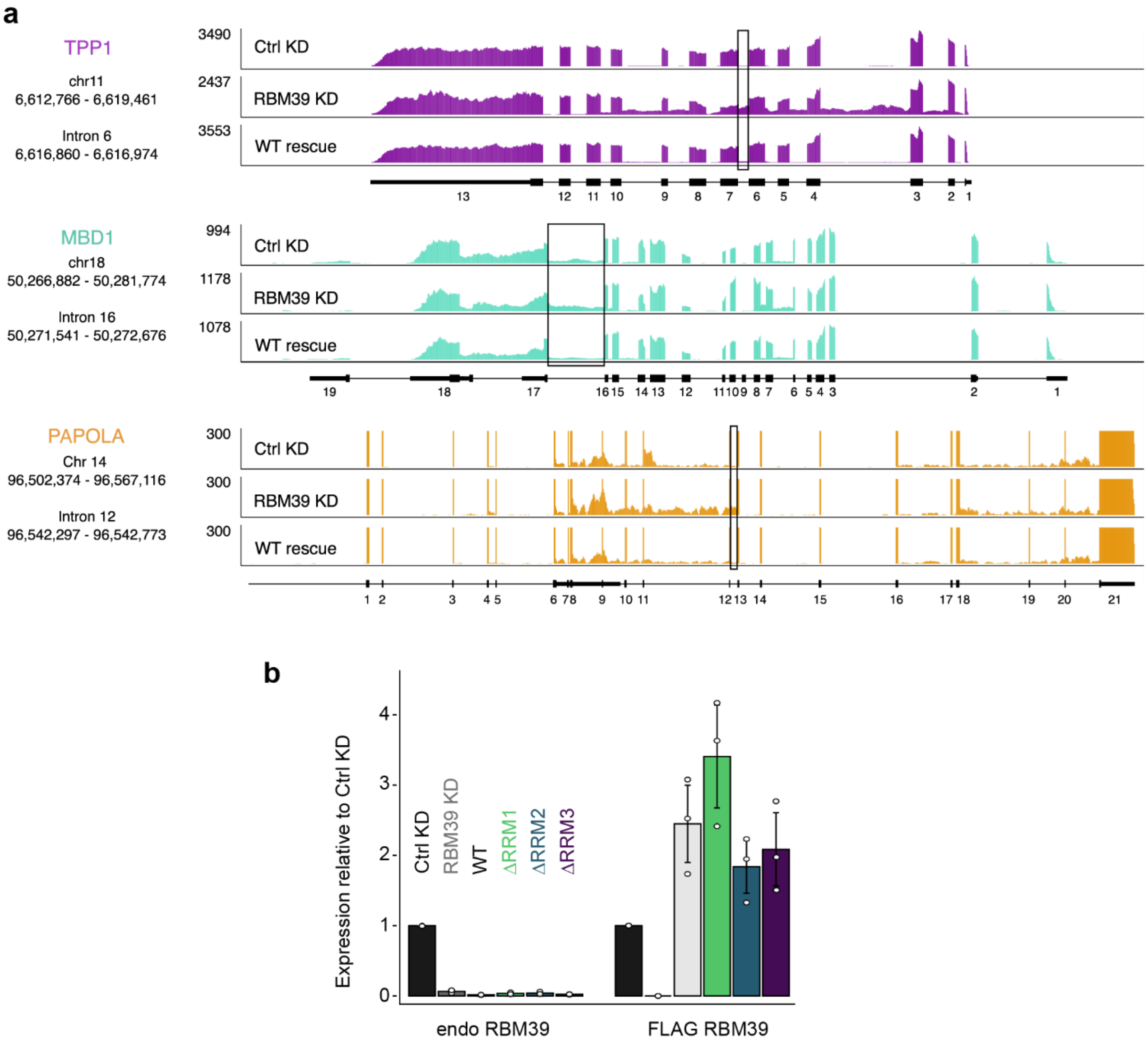

Supplementary Fig. 2: RBM39-dependent intron retention events and protein expression quantification. a, Sashimi plots showing RNA sequencing reads detected for three genes for *TPP1*, *MBD1* and *PAPOLA* upon control (Ctrl KD) or RBM39 knock down (RBM39 KD) and after FLAG-RBM39 rescue (FLAG-RBM39 rescue). In the *TPP1* gene, several intron retention events are RBM39-dependent. **b,** Quantification of the western blot shown in Figure 2a, showing the level of expression of the RBM39 mutants and FLAG-RBM39 mutants relative to the control KD condition. Average values and standard deviations of three biological replicates (n = 3) are shown. P values were computed using two-sided t-test.

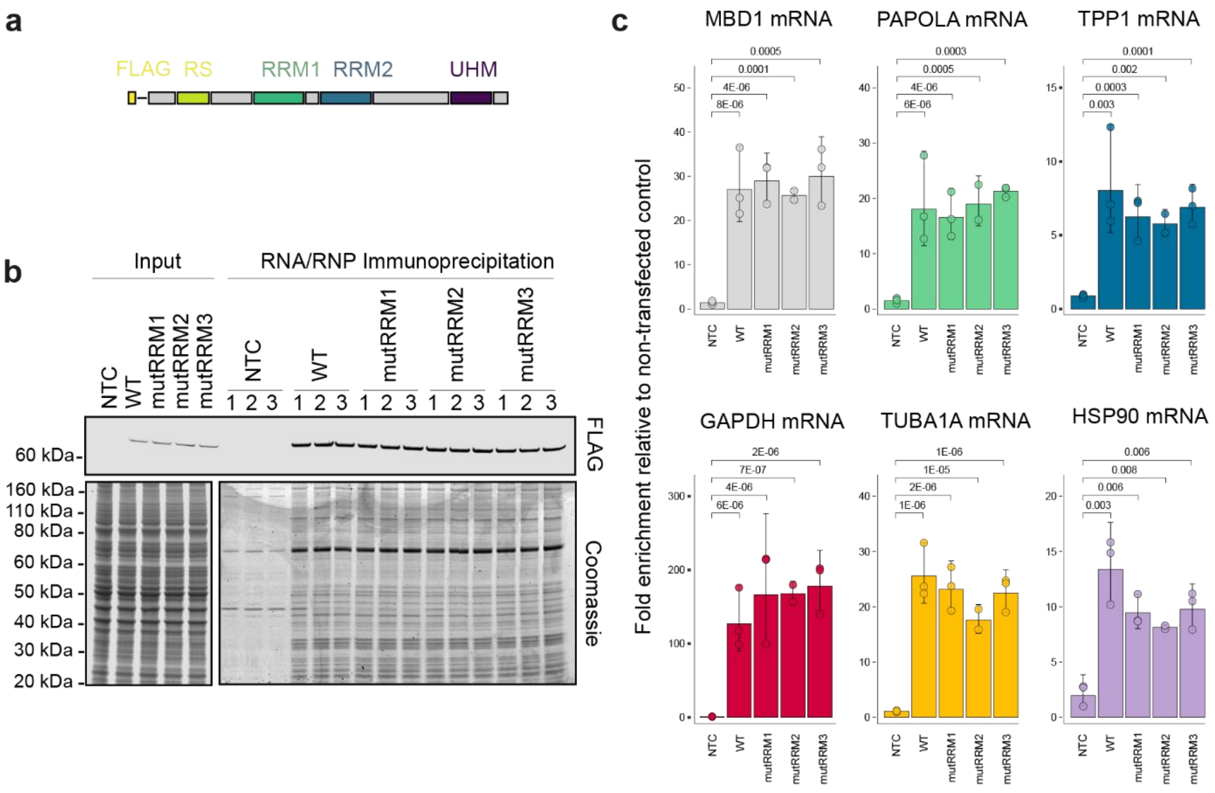

Supplementary Fig. 3: RNA immunoprecipitation using full-length FLAG-RBM39. a, Schematic representation of FLAG-RBM39. **b,** On top, a western blot probed using anti-FLAG antibodies indicates similar expression of the constructs in the input and confirms efficient immunoprecipitation. The SDS-PAGE gel below was stained using Coomassie blue. **c,** Bar plots showing the fold enrichment relative to non-transfected control for the MBD1, PAPOLA, TPP1, GAPDH, TUBA1A and HSP90 mRNAs co-immunoprecipitated with FLAG-RBM39 and its derivatives. Average values and standard deviations of three biological replicates (n = 3) are shown. P values were computed from log-transformed ratios using two-sided unequal variances Welch’s t-test.

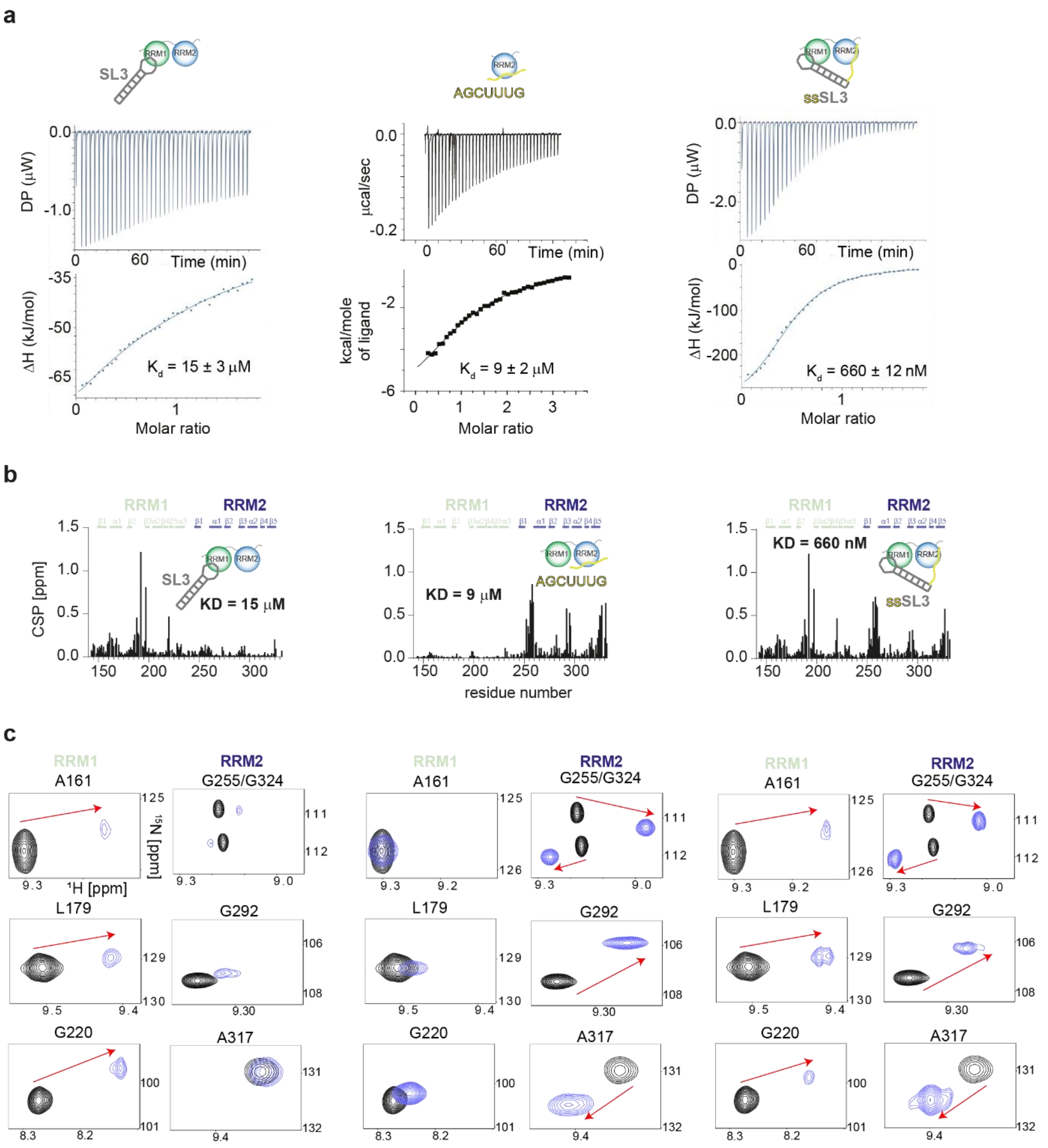

Supplementary Fig. 4: ITC measurements and NMR titrations. a, From left to right, ITC titrations of RRM12 with SL3, RRM2 with AGCUUUG and of RRM12 and ssSL3. Average dissociation constants and errors are given on the plot (n = 3). **b,** Bar plots showing the amide CSPs of RRM12 observed upon addition of either U1 snRNA SL3, the ssRNA motif AGCUUUG or the bipartite RNA motifs AGCUUUG-SL3 (ssSL3). For each NMR titration, the protein was concentrated to 200 μM and the RNA solution stock was at 2 mM. **c,** Portions of the 2D ^{1}H-^{15}N HSQC spectra recorded before (black) and after addition of 1 molar equivalent of RNA (blue).

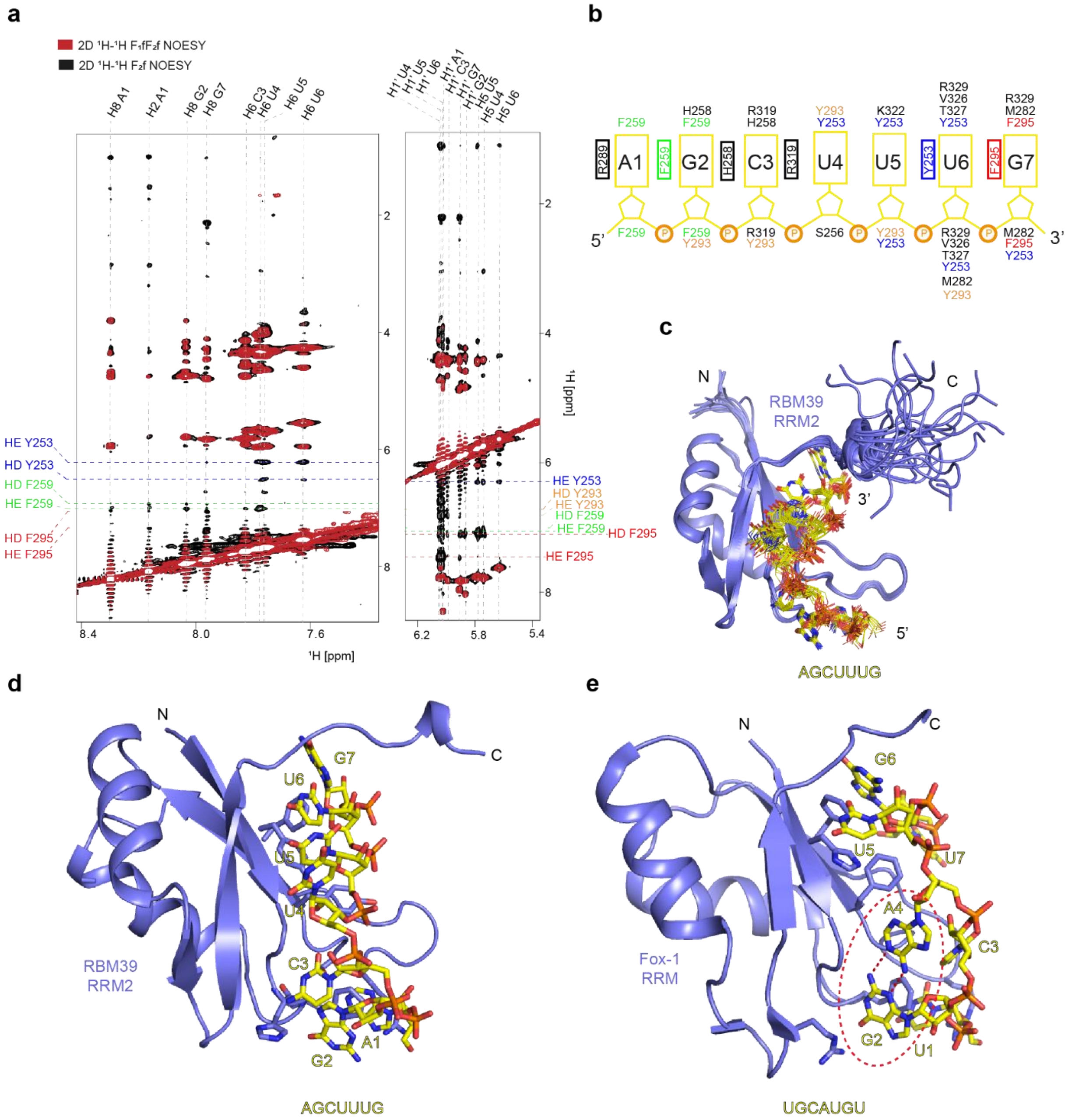

Supplementary Fig. 5: Identification of intermolecular NOEs, solution structure of the RRM2-AGCUUUG complex and comparison with Fox-1 RRM – RNA interaction. a, Overlay of the 2D ^{1}H-^{1}H F1fF2f NOESY and 2D ^{1}H-^{1}H F2f NOESY spectra recorded with a sample of unlabelled AGCUUUG bound to ^{15}N-^{13}C-labelled RRM2. The experiment was recorded with a 1 mM protein sample in presence of 1.25 molar equivalent of RNA. **b,** Schematic showing the intermolecular NOEs observed in the NMR data. **c,** Superimposition of the 20 solution structures of the RRM2 – AGCUUUG complex. **d,** Cartoon representation of the lowest energy model of the NMR structure of the RRM2 – AGCUUUG complex. **e,** Cartoon representation of the lowest energy model of the NMR structure of the Fox-1 RRM2 – UGCAUGU complex (PDB ID 2EER). The formation of the intermolecular base pair between A4 and G2 is highlighted by the red ellipse.

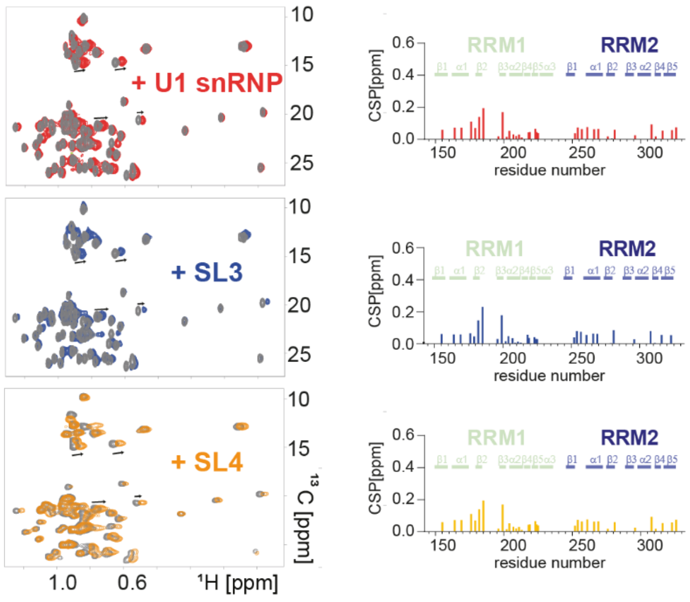

Supplementary Fig. 6: RBM39 interacts with stem loops of U1 snRNP. Overlay of the 2D ^{1}H-^{13}C HMQC spectra of RRM12 isolated (grey) and after addition of 1 molar equivalent of either *in vitro* reconstituted U1 snRNP (red), purified U1 snRNA SL3 (blue) or purified U1 snRNA SL4 (orange). The titrations were performed with a 80 µM protein solution, 1 mM RNA stock solutions and 800 µM U1 snRNP. Bar plots showing the methyl chemical shift perturbations (CSP) observed upon addition of either U1 snRNP, U1 snRNA SL3 or SL4 are displayed.

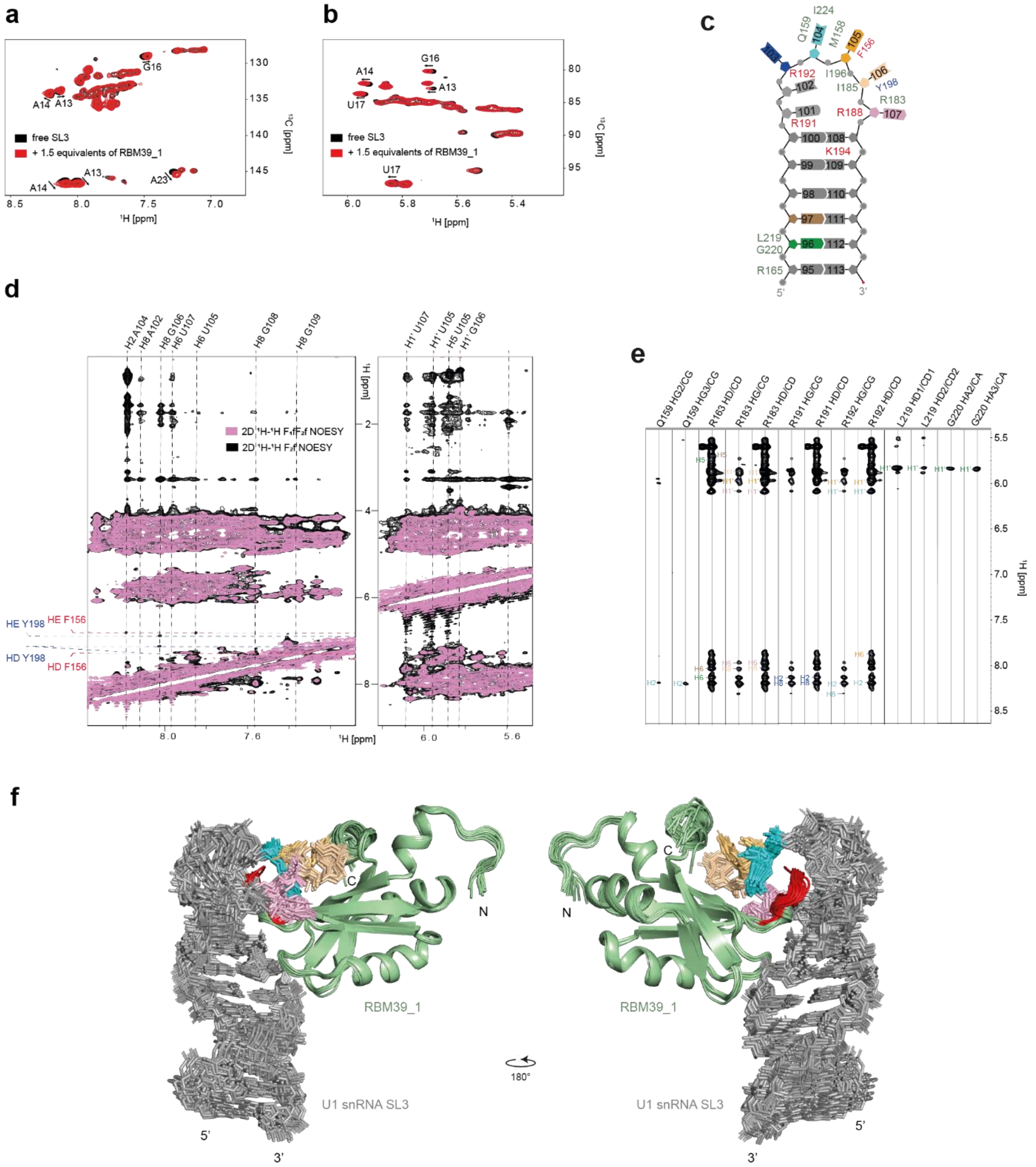

Supplementary Fig. 7: RNA spectra, identification of intermolecular NOEs and solution structure of the RRM1-U1 SL3 complex. a, Overlay of the 2D ^{1}H-^{13}C$_{aromatic}$ HSQC spectra of U1 SL3 before and after addition of unlabelled RRM1. The titration was performed using a 200 µM RNA solution and a 2 mM protein stock solution. **b,** Overlay of the 2D ^{1}H-^{13}C$_{sugar}$ HSQC spectra of U1 SL3 before and after addition of unlabelled RRM1. The titration was performed using a 200 µM RNA solution and a 2 mM protein stock solution. **c,** Scheme showing the intermolecular interactions between RRM1 and SL3. **d,** Overlay of the 2D ^{1}H-^{1}H F1fF2f NOESY and 2D ^{1}H-^{1}H F2f NOESY spectra recorded with a sample of unlabelled U1 SL3 bound to ^{15}N-^{13}C-labelled RRM1. The experiment was recorded with a 1 mM protein sample in presence of 1.25 molar equivalent of RNA. **e,** Selected strips of the 3D ^{13}C-(F1 edited, F3 filtered) NOESY HSQC recorded with ^{13}C-labelled RRM1 and unlabelled SL3. **f,** Superimposition of the 20 solution structures of the RRM1 – SL3 complex.

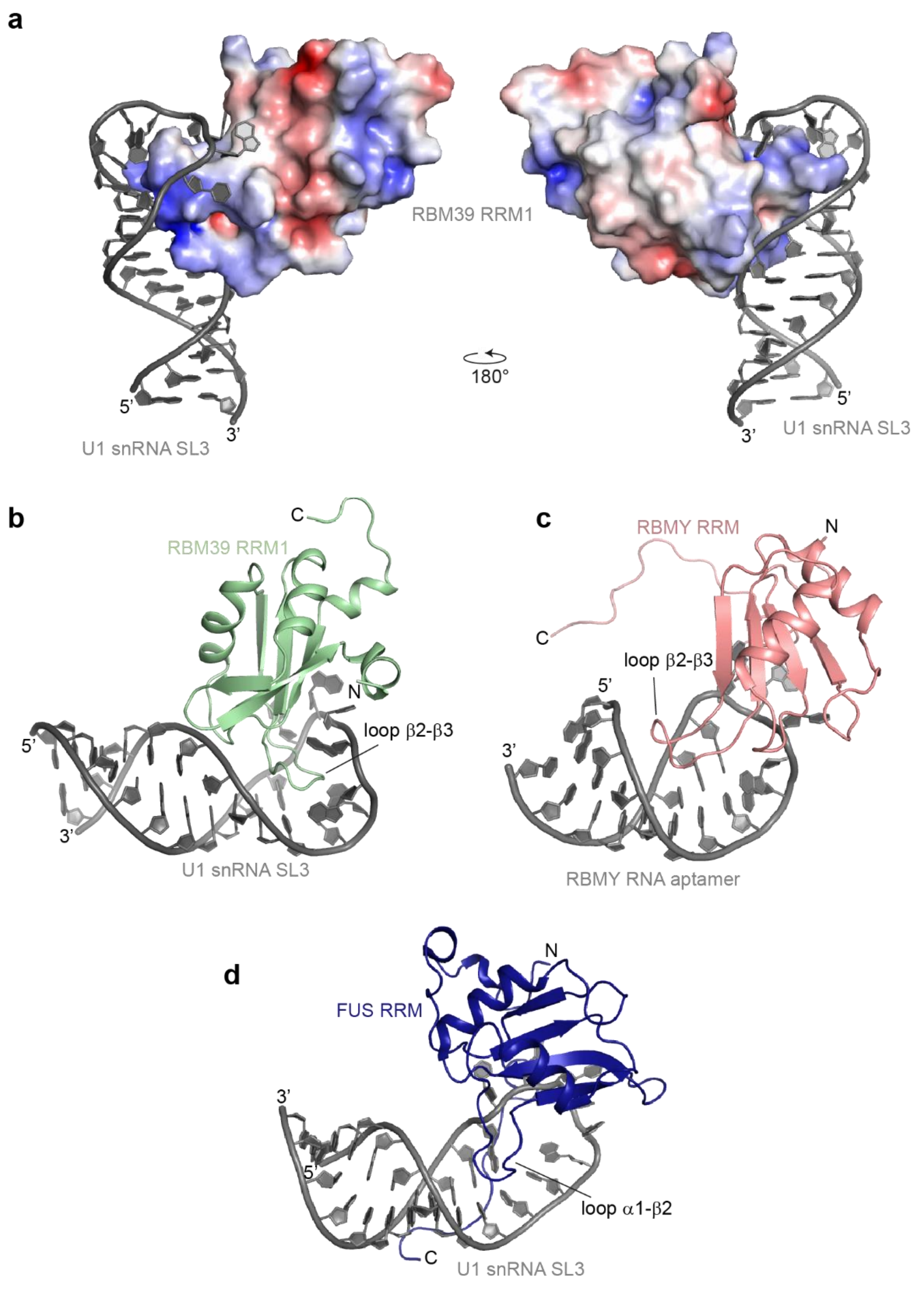

Supplementary Fig. 8: Structural comparison between RBM39, RBMY and FUS RRM interactions with RNA stem loops. a, Electrostatic potential surface of RRM1 when bound to U1 snRNA SL3. **b,** Cartoon representation of the lowest energy model of the NMR structure of RRM1 bound to U1 snRNA SL3. **c,** Cartoon representation of the lowest energy model of the NMR structure of RBMY bound to RBMY RNA aptamer (PDB ID 2FY1). **d,** Cartoon representation of the lowest energy model of the NMR structure of FUS RRM bound to U1 snRNA SL3 (PDB ID 26SNJ).

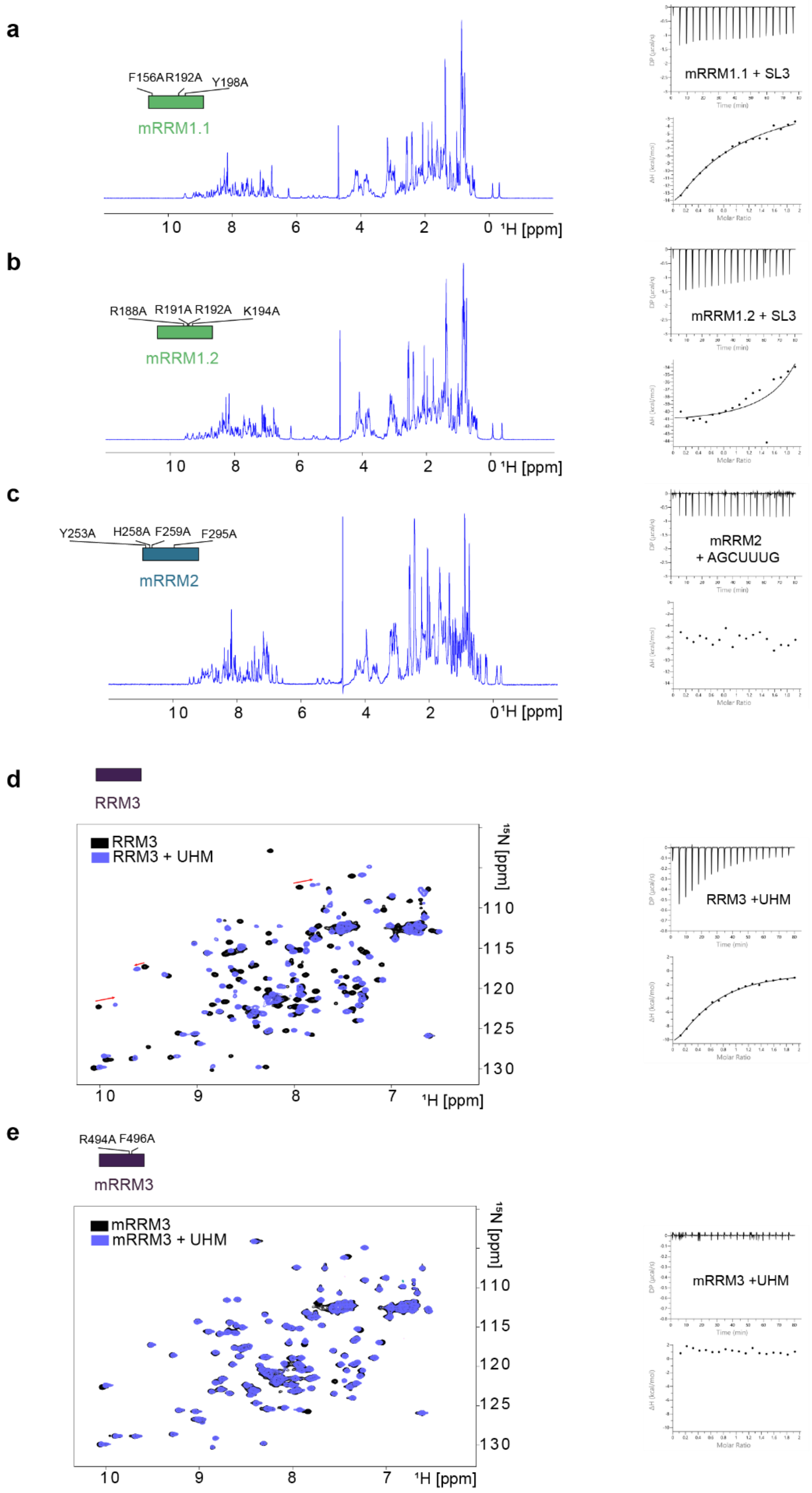

Extended data Fig. 9: Folding and reduced protein or RNA-binding activities of RBM39 mutants. a, 1D 1H NMR spectrum of mRRM1.1 recorded with a protein concentration of 80 µM. On the right, the ITC titration of mRRM1.1 with U1 snRNA SL3 is displayed. **b,** 1D 1H NMR spectrum of mRRM1.2

recorded with a protein concentration of 80 μM. On the right, the ITC titration of mRRM1.2 with U1 snRNA SL3 is displayed. **c,** 1D 1H NMR spectrum of mRRM2 recorded with a protein concentration of 80 μM. On the right, the ITC titration of mRRM1.1 with AGCUUUG is displayed. **d,** Overlay of the 2D ^{1}H-^{15}N HSQC spectra of RBM39 RRM3 before (black) and after addition of 1,5 molar equivalent of the SF3b155 ULM (KSRWDETP). The spectra were recorded with a protein concentration of 160 μM. On the right, the ITC titration of RRM3 with the SF3b155 ULM is displayed. **e,** Overlay of the 2D ^{1}H-^{15}N HSQC spectra of RBM39 mRRM3 before (black) and after addition of 1,5 molar equivalent of the SF3b155 ULM (KSRWDETP). The spectra were recorded with a protein concentration of 160 μM. On the right, the ITC titration of mRRM3 with the SF3b155 ULM is displayed.

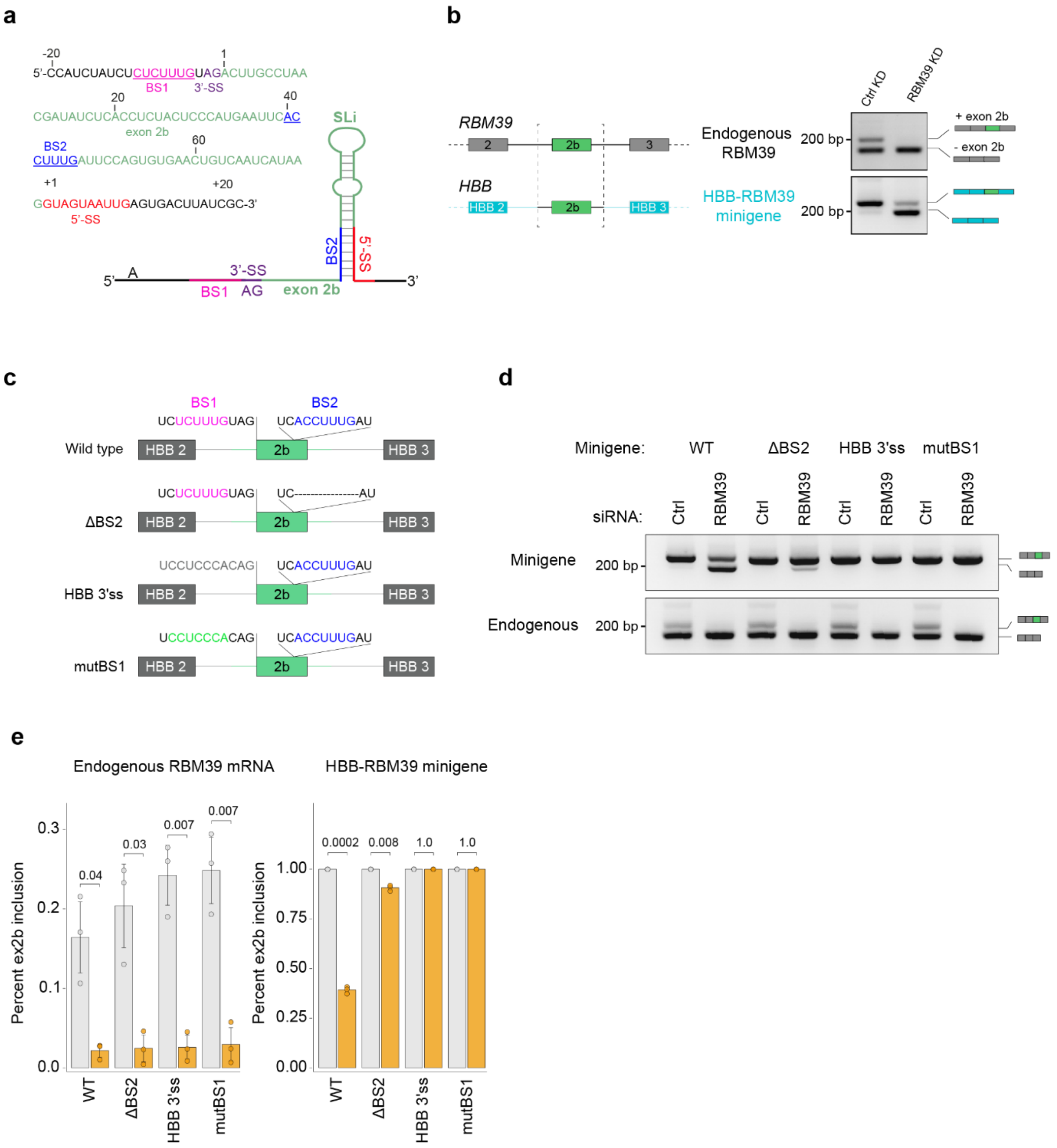

Supplementary Fig. 10: Identification of the *cis* RNA element responsive for the RBM39 dependency of poison exon inclusion in the genetic context of the human β-globin gene. a, Annotated sequence and predicted secondary structures of the *RBM39* exon 2b and its flanking regions. Below, the hybrid minigene containing the exon 2b in the context of the β-globin gene and the different constructs (WT, ΔBS2, HBB 3'ss and mutBS1) are schematically depicted. **b,** Effect of the replacement of the RBM39 poison exon in the genomic context of the β-globin. The splicing of the poison exon was evaluated by RT-PCR in both context upon control KD or RBM39 KD. This experiment was repeated three times and led to similar results. **c,** Schematic representations of the different minigene constructs (WT, ΔBS2, HBB 3'ss and mutBS1). **d,** Agarose gel showing the results of the RT-PCR using the different minigene constructs (WT, ΔBS2, HBB 3'ss and mutBS1) in control and RBM39 KDs. The splicing of the endogenous RBM39 RNA is also displayed for each condition. **e,** Plot showing the percentage of poison exon inclusion was determined by RT-PCR for the different constructs upon Ctlr KD and RBM39 KD. Average values and standard deviations of three biological replicates (n = 3) are shown. P values were computed using two-sided t-test.

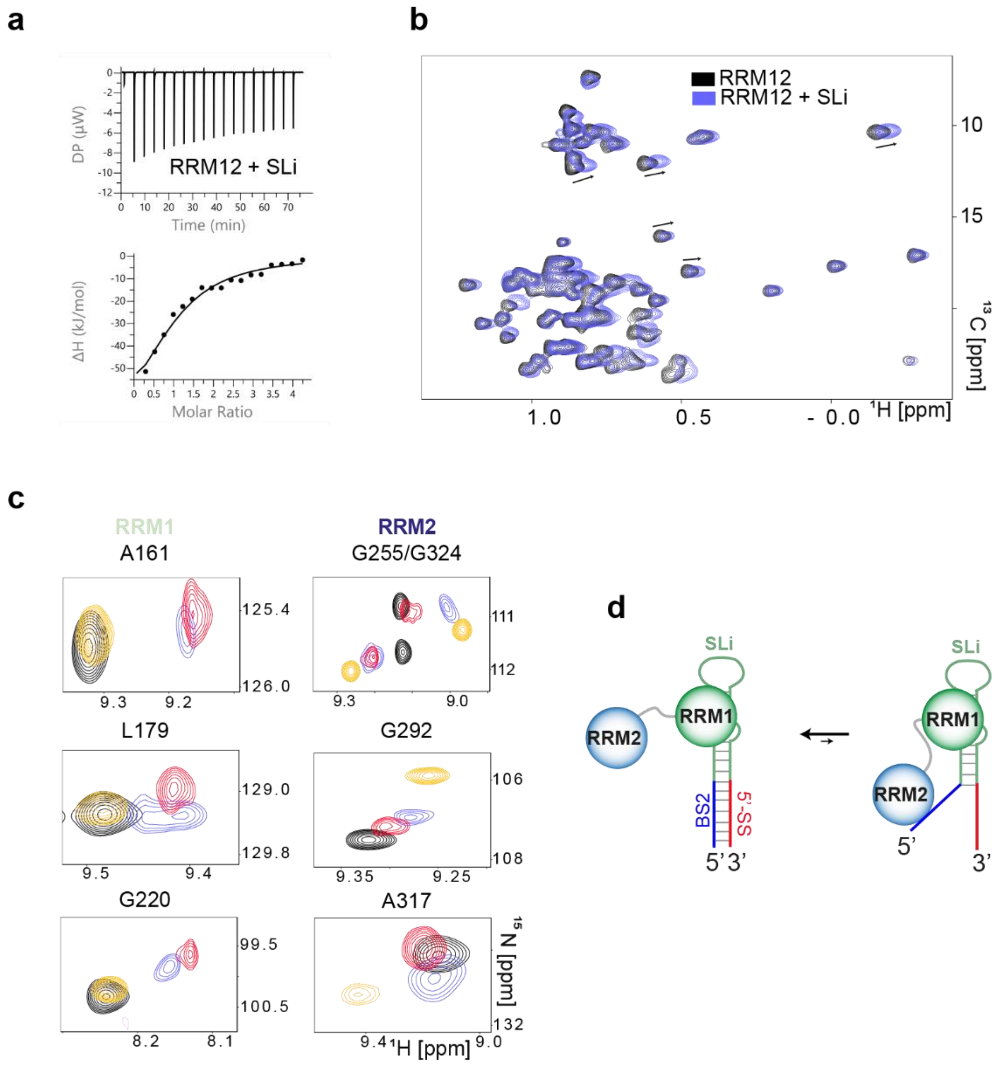

Supplementary Fig. 11: Probing the interaction between RBM39 RRM12 and SLi. a, ITC titration of RBM39 RRM12 and SLi. **b,** Overlay of the 2D ^{1}H-^{13}C HMQC spectra of RRM12 isolated (black) and after addition of 1 molar equivalent of SLi (blue). The titration was performed with a 80 µM protein solution and 1 mM RNA stock solution. **c,** Overlay of portions of the 2D 2D ^{1}H-^{15}N HSQC spectra of RBM39 before (black) and after addition of 1,5 molar equivalent of either SL3 (red), AGCUUUG (yellow) or SLi (blue). All titrations were performed using a protein concentration of 200 µM and 2 mM RNA solution stocks. **d,** Schematic showing the binding of RBM39 RRM12 to SLi *in vitro*. The scheme explains the line broadening effect observed during the titration by a potential mechanism of chemical exchange. The binding of RRM2 to BS2 will compete with the formation of a secondary structure.

Supplementary Table 1 - NMR and refinement statistics for RBM39 RRM1 in complex with SL3

	RRM1	SL3
**NMR distance and dihedral constraints**		
Distance restraints		
Total NOE	2,200	319
Intra-residue	457	131
Inter-residue	1,743	188
Sequential ($\|i – j\| = 1$)	561	126
Nonsequential ($\|i – j\| > 1$)	1,182	52
Hydrogen bonds	9	10
Protein–nucleic acid intermolecular	66	
Total dihedral angle restraints		
Protein		
ϕ	72	
ψ	72	
Nucleic acid		
Base pair		10
Sugar pucker		84
Backbone		56
**Structure statistics**		
Violations (mean and s.d.)		
Distance constraints (Å) > 0.4 Å	2.0 ± 1.2	
Dihedral angle constraints (º) > 5°	2.5 ± 10.9	
Max. dihedral angle violation (º)	6.09 ± 1.34	
Max. distance constraint violation (Å)	0.51 ± 0.19	
Deviations from idealized geometry		
Bond lengths (Å)	0.0038 ± 0.0013	
Bond angles (º)	1.452 ± 0.484	
Average pairwise r.m.s. deviation** (Å)		
Protein		
Heavy	0.40 ± 0.14	
Backbone	0.86 ± 0.17	
RNA		
All RNA heavy		0.79 ± 0.22
RNA binding site		0.82 ± 0.20
Complex		
All complex heavy (C, N, O, P)	0.68 ± 0.17	
Protein and nucleic acid heavy	0.92 ± 0.17	

** RMSD range calculated with the 20 structures of the NMR ensemble with the following residue ranges: 143-283
for the protein and 94-118 for the RNA.

Supplementary Table 2 - NMR and refinement statistics for RBM39 RRM2 in complex with 5'-AGCUUUG-3'

	RRM2	AGCUUUG
**NMR distance and dihedral constraints**		
Distance restraints		
Total NOE	2,214	58
Intra-residue	427	50
Inter-residue	1,787	8
Sequential ($\|i - j\| = 1$)	526	8
Nonsequential ($\|i - j\| > 1$)	1,261	0
Hydrogen bonds	32	
Protein–nucleic acid intermolecular	84	
Total dihedral angle restraints		
Protein		
ϕ	75	
ψ	75	
Nucleic acid		
Base pair		0
Sugar pucker		0
Backbone		0
**Structure statistics**		
Violations (mean and s.d.)		
Distance constraints (Å) > 0.4 Å	2.6 ± 1.4	
Dihedral angle constraints (º) > 5°	2.5 ± 0.9	
Max. dihedral angle violation (º)	6.02 ± 3.96	
Max. distance constraint violation (Å)	0.52 ± 0.19	
Deviations from idealized geometry		
Bond lengths (Å)	0.0041 ± 0.0001	
Bond angles (º)	1.404 ± 0.008	
Average pairwise r.m.s. deviation** (Å)		
Protein		
Heavy	0.20 ± 0.04	
Backbone	0.45 ± 0.06	
RNA		
All RNA heavy		0.72 ± 0.16
RNA binding site		0.71 ± 0.15
Complex		
All complex heavy (C, N, O, P)	0.71 ± 0.16	
Protein and nucleic acid heavy	0.79 ± 0.15	

** RMSD range calculated with the 20 structures of the NMR ensemble with the following residue ranges: 284-370
for the protein and 1-7 for the RNA.
