# Supplementary material for: Molecular basis of RNA-binding and autoregulation by the cancer-associated splicing factor RBM39
Source: Nat Commun. 2023 Sep 4;14:5366. doi: 10.1038/s41467-023-40254-5 (PMC10477243; doi:10.1038/s41467-023-40254-5)
Supplement: Supplementary file 3 — Description of Additional Supplementary Files [file 41467_2023_40254_MOESM3_ESM.pdf]

Description of Additional Supplementary Files

File Name: Supplementary Movie 1
Description: Structure of RBM39 RRM2 bound to 5'-AGCUUUG-3'

File Name: Supplementary Movie 2
Description: Structure of RBM39 RRM1 bound to U1 snRNA stem loop 3

File Name: Supplementary Data 1
Description: Oligonucleotide list
