# Supplementary material for: Molecular basis of RNA-binding and autoregulation by the cancer-associated splicing factor RBM39
Source: Nat Commun. 2023 Sep 4;14:5366. doi: 10.1038/s41467-023-40254-5 (PMC10477243; doi:10.1038/s41467-023-40254-5)
Supplement: Supplementary file 7 — Reporting Summary [file 41467_2023_40254_MOESM7_ESM.pdf]

nature portfolio

Corresponding author(s): Frédéric HT Allain
Marc-David Ruepp
Sébastien Campagne

Last updated by author(s): 2023/06/28

Reporting Summary

Nature Portfolio wishes to improve the reproducibility of the work that we publish. This form provides structure for consistency and transparency in reporting. For further information on Nature Portfolio policies, see our Editorial Policies and the Editorial Policy Checklist.

Statistics

For all statistical analyses, confirm that the following items are present in the figure legend, table legend, main text, or Methods section.

n/a	Confirmed	
☐	☒	The exact sample size (*n*) for each experimental group/condition, given as a discrete number and unit of measurement
☐	☒	A statement on whether measurements were taken from distinct samples or whether the same sample was measured repeatedly
☐	☒	The statistical test(s) used AND whether they are one- or two-sided<br>*Only common tests should be described solely by name; describe more complex techniques in the Methods section.*
☒	☐	A description of all covariates tested
☒	☐	A description of any assumptions or corrections, such as tests of normality and adjustment for multiple comparisons
☐	☒	A full description of the statistical parameters including central tendency (e.g. means) or other basic estimates (e.g. regression coefficient) AND variation (e.g. standard deviation) or associated estimates of uncertainty (e.g. confidence intervals)
☐	☒	For null hypothesis testing, the test statistic (e.g. *F*, *t*, *r*) with confidence intervals, effect sizes, degrees of freedom and *P* value noted<br>*Give P values as exact values whenever suitable.*
☒	☐	For Bayesian analysis, information on the choice of priors and Markov chain Monte Carlo settings
☒	☐	For hierarchical and complex designs, identification of the appropriate level for tests and full reporting of outcomes
☒	☐	Estimates of effect sizes (e.g. Cohen's *d*, Pearson's *r*), indicating how they were calculated

Our web collection on statistics for biologists contains articles on many of the points above.

Software and code

Policy information about availability of computer code

Data collection	All software is publicly or commercially available. See methods section for literature references.<br>To collect the data, we used the following software:<br>Western blot: Image Studio (LI-COR Biosciences, v5.2)<br>RT-qPCR: Rotor-Gene Q software (Qiagen, 2.1.0.9)<br>Microscopy: NIS elements AR v5.01 (Nikon), Leica Application Suite X (LAS-X).<br>NMR: Topspin3.2 (Bruker)<br>ITC: Origin 7.0, PEAQ-ITC (Malvern)
Data analysis	All software is publicly or commercially available. To analyse the data, we used the following softwares:<br>Western blot: Image Studio (LI-COR Biosciences, v5.2)<br>Microscopy: NIS elements AR v5.01 (Nikon), Leica Application Suite X (LAS-X), Fiji v2.0.0<br>RT-qPCR: Microsoft Excel (v16.72), RStudio (Posit PBC)<br>RNA-Seq: STAR aligner version 2.5.2a, Subread package (version 1.4.6), DESeq2 version 1.6.3, DEXseq.<br>NMR: CARA v1.9, UNIO–ATNOS–CANDID, CYANA 3.9, TALOS+, AMBER20, PROCHECK.<br>ITC: Origin 7.0, PEAQ-ITC (Malvern)<br>In addtion, we also used custom scripts for the analysis of intron retention that were deposited in GitHub: https://github.com/Martombo/SpliceRatio. A code availability statement was added in the manuscript.

For manuscripts utilizing custom algorithms or software that are central to the research but not yet described in published literature, software must be made available to editors and reviewers. We strongly encourage code deposition in a community repository (e.g. GitHub). See the Nature Portfolio guidelines for submitting code & software for further information.

Data

Policy information about availability of data

All manuscripts must include a data availability statement. This statement should provide the following information, where applicable:

- Accession codes, unique identifiers, or web links for publicly available datasets
- A description of any restrictions on data availability
- For clinical datasets or third party data, please ensure that the statement adheres to our policy

The RNA-seq data have been deposited into the GEO database under the accession code GSE202134 (https://www.ncbi.nlm.nih.gov/geo/query/acc.cgi?acc=GSE202134). The atomic coordinates of the structure of the RRM1-SL3 complex have been deposited in the PDB under the accession code 7ZAP (https://doi.org/10.2210/pdb7ZAP/pdb). The chemical shifts of the RRM1-SL3 complex were deposited in the BMRB under the accession code 34715 (https://doi.org/10.13018/BMR34715). The atomic coordinates of the structure of the RRM2-AGCUUUG complex have been deposited in the PDB under the accession code 7Q33 (https://doi.org/10.2210/pdb7Q33/pdb). The chemical shifts of the RRM2-AGCUUUG complex were deposited in the BMRB under the accession code 34673 (https://doi.org/10.13018/BMR34673).

Human research participants

Policy information about studies involving human research participants and Sex and Gender in Research.

Reporting on sex and gender	N/A
Population characteristics	N/A
Recruitment	N/A
Ethics oversight	N/A

Note that full information on the approval of the study protocol must also be provided in the manuscript.

Field-specific reporting

Please select the one below that is the best fit for your research. If you are not sure, read the appropriate sections before making your selection.

☒ Life sciences ☐ Behavioural & social sciences ☐ Ecological, evolutionary & environmental sciences

For a reference copy of the document with all sections, see nature.com/documents/nr-reporting-summary-flat.pdf

Life sciences study design

All studies must disclose on these points even when the disclosure is negative.

Sample size	No sample size calculation was performed. All experiments were performed at least in triplicate, which is common practice for molecular biology experiments. We consider this sufficient since the observed effect sizes were large in comparison to the variability between replicates.
Data exclusions	No data was excluded.
Replication	All experiments described in this study were successfully reproduced at least three times and n numbers are provided in the figure legends. All experimental parameters required for the reproduction by other labs are provided in the methods section. All attempts at replication were successful.
Randomization	Randomisation was not applicable as samples were not assigned to experimental groups
Blinding	Blinding was not relevant for this study as no subjective rating (e.g manual counting of features) was performed. Quantitative measurements were performed by machines and the effects described in qualitative experiments were obvious and of large size

Behavioural & social sciences study design

All studies must disclose on these points even when the disclosure is negative.

Study description	N/A

Research sample	N/A
Sampling strategy	N/A
Data collection	N/A
Timing	N/A
Data exclusions	N/A
Non-participation	N/A
Randomization	N/A

Ecological, evolutionary & environmental sciences study design

All studies must disclose on these points even when the disclosure is negative.

Study description	N/A
Research sample	N/A
Sampling strategy	N/A
Data collection	N/A
Timing and spatial scale	N/A
Data exclusions	N/A
Reproducibility	N/A
Randomization	N/A
Blinding	N/A

Did the study involve field work? ☐ Yes ☐ No

Field work, collection and transport

Field conditions	N/A
Location	N/A
Access & import/export	N/A
Disturbance	N/A

Reporting for specific materials, systems and methods

We require information from authors about some types of materials, experimental systems and methods used in many studies. Here, indicate whether each material, system or method listed is relevant to your study. If you are not sure if a list item applies to your research, read the appropriate section before selecting a response.

Materials & experimental systems

n/a	Involved in the study
☐	☒ Antibodies
☐	☒ Eukaryotic cell lines
☒	☐ Palaeontology and archaeology
☒	☐ Animals and other organisms
☒	☐ Clinical data
☒	☐ Dual use research of concern

Methods

n/a	Involved in the study
☒	☐ ChIP-seq
☒	☐ Flow cytometry
☒	☐ MRI-based neuroimaging

Antibodies

Antibodies used

Mouse anti-FLAG M2 (Sigma, F1804), mouse anti-SmB/B′ (Y12, Lerner et al 1981; Protein G purified from supernatant of hybridoma cells, kind gift from Daniel Schümperli, University of Bern; Antibody is also commercially available e.g. from Invitrogen (MA5-13449), or Merck (MABF2793-100UL)), mouse anti-U1A (SCBT, sc-101149), rabbit anti-RBM39 (Bethyl, A300-291A), mouse anti-GAPDH (SCBT, sc-32233), mouse anti-TUB-1A2 (Sigma, T9028), mouse IgG (Jackson Immuno Research, 015-000-003), rabbit IgG (SCBT, sc-2027), rabbit anti-U1C (Bethyl, A303-947A), rabbit anti-RBM39 (Sigma HPA001591), mouse anti-SF3A3 (SCBT, sc-374464), Chicken anti-Mouse AF488 (Invitrogen, A-21200), donkey anti-mouse IRDye800CW (LI-COR Biosciences, 926- 32212) and donkey anti-rabbit IRDye800CW (LI-COR Biosciences, 926-32213), Mouse IgG (SCBT, sc-2025), Rabbit IgG (Jackson Immuno Research, 011-000-003), Rabbit anti-U1C (Abcam, ab192028), rabbit anti-Actin (Sigma-Aldrich, A5060), goat anti-UPF1 (Bethyl Laboratories, A300-038A).

Validation

Mouse anti-FLAG M2: https://www.sigmaaldrich.com/GB/en/product/sigma/f3165. Validated by overexpression of tagged constructs and >8900 citations covering WB, IF, IP
Mouse anti-SmB/B′ (Y12): Characterized in original publication: Lerner et al. 1981. Proc Natl Acad Sci U S A. 78(5):2737-41. PMID: 6789322.
Mouse anti-U1A: https://datasheets.scbt.com/sc-101149.pdf. Validated by manufacturer for WB: correct size, IF: correct cellular localisation.
Rabbit anti-RBM39: https://www.fortislife.com/cms/files/A300-291A-3.pdf. Validated by manufacturer for WB: correct size, IHC: correct cellular localisation, IP: Correct size
Mouse anti-GAPDH: https://datasheets.scbt.com/sc-32233.pdf. Validated by manufacturer for WB: Correct size, IF: Correct cellular localisation
Mouse anti-TUB-1A2: https://www.sigmaaldrich.com/deepweb/assets/sigmaaldrich/product/documents/334/636/sab4200776dat.pdf Fully chacterised in original publication (Kreis TE., EMBO J., 6, 2597-606 (1987)). Recognises a single band at the expected molecular weight.
Mouse IgG: https://www.jacksonimmuno.com/catalog/products/015-000-003. Control IgG from non-immunised animals
Rabbit IgG: https://datasheets.scbt.com/sc-2027.pdf Control IgG from non-immunised animals
Rabbit anti-U1C: Validated by manufacturer for Western blot and IP
Rabbit anti-RBM39: https://www.sigmaaldrich.com/GB/en/product/sigma/hpa001591. Validated by manufacturer for WB: correct size and via knockdown.
Mouse anti-SF3A3: https://datasheets.scbt.com/sc-374464.pdf Validated by manufacturer for WB: Correct size, IF: Correct cellular localisation
Chicken anti-Mouse AF488: https://www.thermofisher.com/antibody/product/Chicken-anti-Mouse-IgG-H-L-Cross-Adsorbed-Secondary-Antibody-Polyclonal/A-21200. Validated by manufacturer for IF
Donkey anti-mouse IRDye800CW: https://www.licor.com/documents/ri8oae8r69uvqndq72g3l9a4d0do4u44. Tested and qualified for WB by manufacturer
Donkey anti-rabbit IRDye800CW: https://www.licor.com/documents/ri8oae8r69uvqndq72g3l9a4d0do4u44. Tested and qualified for WB by manufacturer
Mouse IgG: https://www.scbt.com/de/p/normal-mouse-igg. Control IgG from non-immunised animals
Rabbit IgG: https://www.jacksonimmuno.com/catalog/products/011-000-003. Control IgG from non-immunised animals
Rabbit anti-U1C (Bethyl, A303-947A): Product has been discontinued by the manufacturer. Antibody has been used for WB in the following publications, where a band corresponding to the expected molecular weight is present in purified spliceosomal complexes: 10.1016/j.celrep.2016.05.002 and 10.1016/j.molcel.2018.09.004 .
Rabbit anti-U1C (Abcam, ab192028): https://www.abcam.com/products/primary-antibodies/u1-c-antibody-epr16034-ab192028.html. Validated by manufacturer for WB: Correct size, IP: Correct size, IF: Correct cellular localisation.
Rabbit anti-Actin: https://www.sigmaaldrich.com/GB/en/product/sigma/a5060?%C2%AEion=US. Validated by manufacturer for WB: Correct size, IF: Signal overlaps completely with an unrelated Actin antibody, IHC: Correct cellular localisation.
Goat anti-UPF1: https://www.thermofisher.com/antibody/product/RENT1-Antibody-Polyclonal/A300-038A. Validated by manufacturer for WB: Correct size, IP: Correct size.

Eukaryotic cell lines

Policy information about cell lines and Sex and Gender in Research

Cell line source(s)

The HeLa cells (ATCC CLL-2) were a gift from Oliver Muehlemann (University of Bern)

Authentication

HeLa cell stocks were authenticated through STR profiling by Microsynth (Balgach, Switzerland). The cells match 93.8% to the DNA profile of the ATCC CLL-2 cell line.

Mycoplasma contamination

Cells tested negative for mycoplasma

Commonly misidentified lines (See ICLAC register)	No commonly misidentified cell lines were used in this study.

Palaeontology and Archaeology

Specimen provenance	N/A
Specimen deposition	N/A
Dating methods	N/A

☐ Tick this box to confirm that the raw and calibrated dates are available in the paper or in Supplementary Information.

Ethics oversight	N/A

Note that full information on the approval of the study protocol must also be provided in the manuscript.

Animals and other research organisms

Policy information about studies involving animals; ARRIVE guidelines recommended for reporting animal research, and Sex and Gender in Research

Laboratory animals	N/A
Wild animals	N/A
Reporting on sex	N/A
Field-collected samples	N/A
Ethics oversight	N/A

Note that full information on the approval of the study protocol must also be provided in the manuscript.

Clinical data

Policy information about clinical studies

All manuscripts should comply with the ICMJE guidelines for publication of clinical research and a completed CONSORT checklist must be included with all submissions.

Clinical trial registration	N/A
Study protocol	N/A
Data collection	N/A
Outcomes	N/A

Dual use research of concern

Policy information about dual use research of concern

Hazards

Could the accidental, deliberate or reckless misuse of agents or technologies generated in the work, or the application of information presented in the manuscript, pose a threat to:

No	Yes	
☒	☐	Public health
☒	☐	National security
☒	☐	Crops and/or livestock
☒	☐	Ecosystems
☒	☐	Any other significant area

Experiments of concern

Does the work involve any of these experiments of concern:

No	Yes	
☒	☐	Demonstrate how to render a vaccine ineffective
☒	☐	Confer resistance to therapeutically useful antibiotics or antiviral agents
☒	☐	Enhance the virulence of a pathogen or render a nonpathogen virulent
☒	☐	Increase transmissibility of a pathogen
☒	☐	Alter the host range of a pathogen
☒	☐	Enable evasion of diagnostic/detection modalities
☒	☐	Enable the weaponization of a biological agent or toxin
☒	☐	Any other potentially harmful combination of experiments and agents

ChIP-seq

Data deposition

☐ Confirm that both raw and final processed data have been deposited in a public database such as GEO.

☐ Confirm that you have deposited or provided access to graph files (e.g. BED files) for the called peaks.

Data access links<br>*May remain private before publication.*	N/A
Files in database submission	N/A
Genome browser session<br>(e.g. UCSC)	N/A

Methodology

Replicates	N/A
Sequencing depth	N/A
Antibodies	N/A
Peak calling parameters	N/A
Data quality	N/A
Software	N/A

Flow Cytometry

Plots

Confirm that:

☐ The axis labels state the marker and fluorochrome used (e.g. CD4-FITC).

☐ The axis scales are clearly visible. Include numbers along axes only for bottom left plot of group (a 'group' is an analysis of identical markers).

☐ All plots are contour plots with outliers or pseudocolor plots.

☐ A numerical value for number of cells or percentage (with statistics) is provided.

Methodology

Sample preparation	N/A
Instrument	N/A
Software	N/A
Cell population abundance	N/A

Gating strategy: N/A

☐ Tick this box to confirm that a figure exemplifying the gating strategy is provided in the Supplementary Information.

Magnetic resonance imaging

Experimental design

Design type	N/A
Design specifications	N/A
Behavioral performance measures	N/A

Acquisition

Imaging type(s)	N/A
Field strength	N/A
Sequence & imaging parameters	N/A
Area of acquisition	N/A

Diffusion MRI ☐ Used ☐ Not used

Preprocessing

Preprocessing software	N/A
Normalization	N/A
Normalization template	N/A
Noise and artifact removal	N/A
Volume censoring	N/A

Statistical modeling & inference

Model type and settings	N/A
Effect(s) tested	N/A

Specify type of analysis: ☐ Whole brain ☐ ROI-based ☐ Both

Statistic type for inference (See Eklund et al. 2016)	N/A
Correction	N/A

Models & analysis

n/a	Involved in the study
☒	☐ Functional and/or effective connectivity
☒	☐ Graph analysis
☒	☐ Multivariate modeling or predictive analysis

Functional and/or effective connectivity	*Report the measures of dependence used and the model details (e.g. Pearson correlation, partial correlation, mutual information).*
Graph analysis	*Report the dependent variable and connectivity measure, specifying weighted graph or binarized graph, subject- or group-level, and the global and/or node summaries used (e.g. clustering coefficient, efficiency, etc.).*
Multivariate modeling and predictive analysis	*Specify independent variables, features extraction and dimension reduction, model, training and evaluation metrics.*
