# Supplementary material for: Molecular basis of RNA-binding and autoregulation by the cancer-associated splicing factor RBM39
Source: Nat Commun. 2023 Sep 4;14:5366. doi: 10.1038/s41467-023-40254-5 (PMC10477243; doi:10.1038/s41467-023-40254-5)

Supplementary Figure 1c

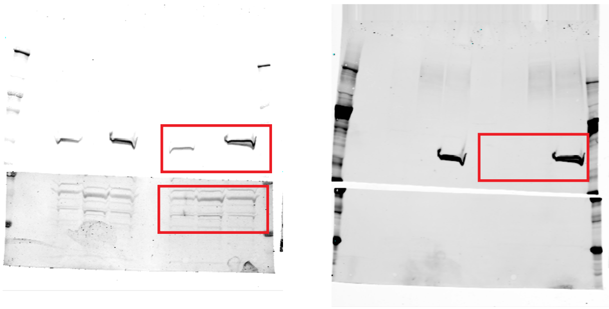

Supplementary Figure 1g

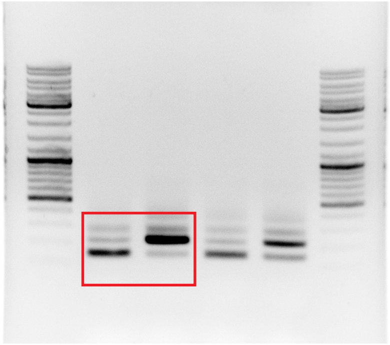

Supplementary Figure 1e

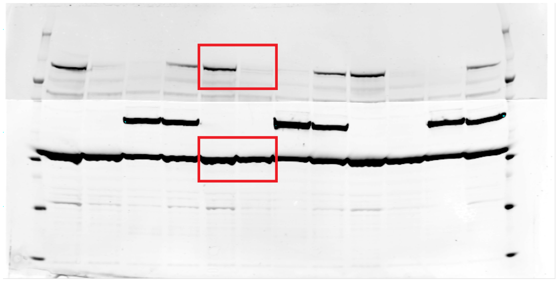

Supplementary Figure 3b

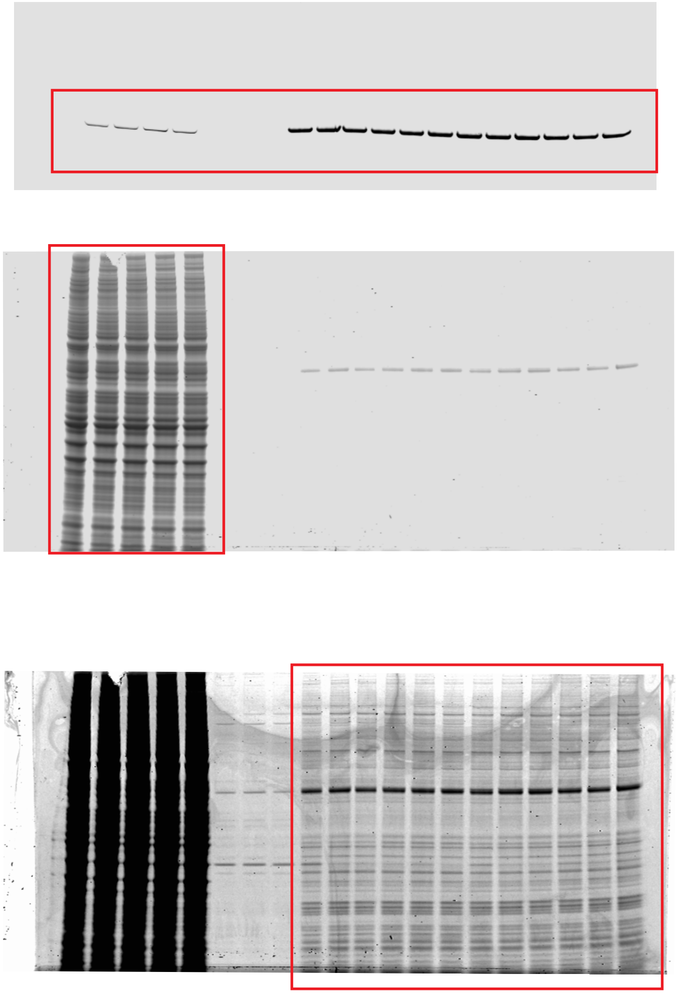

Supplement: Supplementary file 8 — Source data [file 41467_2023_40254_MOESM8_ESM.zip › Source data/Uncropped_gels_SuppFig1_3.pdf]
