# Supplementary figures and images for: Molecular basis of RNA-binding and autoregulation by the cancer-associated splicing factor RBM39
Source: Nat Commun. 2023 Sep 4;14:5366. doi: 10.1038/s41467-023-40254-5 (PMC10477243; doi:10.1038/s41467-023-40254-5)

Fig. 1f

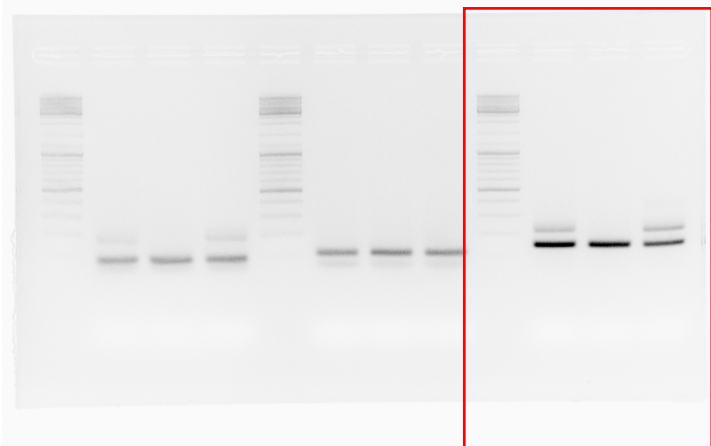

Fig. 2a

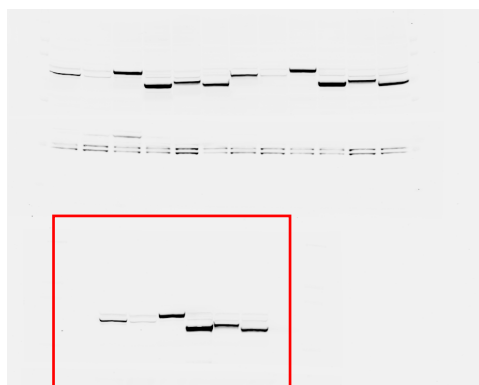

Fig. 2e

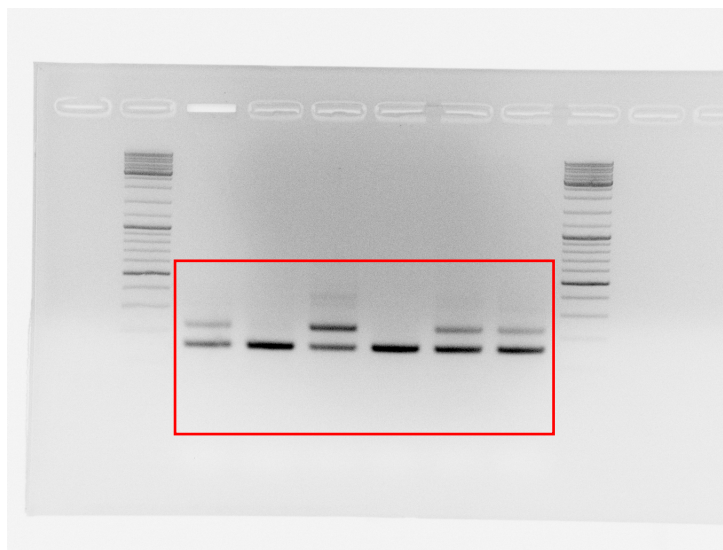

Fig. 2a

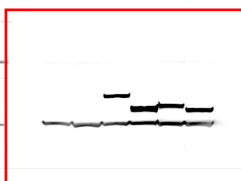

Supplement: Supplementary file 8 — Source data [file 41467_2023_40254_MOESM8_ESM.zip › Source data/Uncropped_gels_Fig1_2.pdf]

Fig. 3b

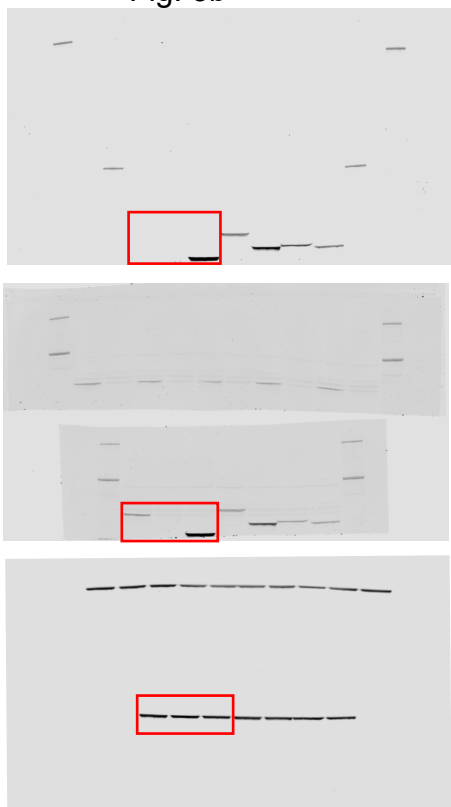

Fig. 3f

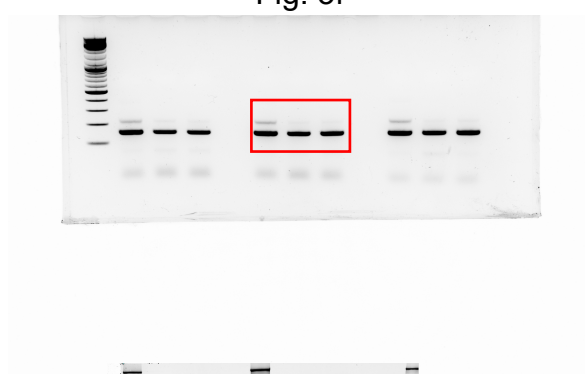

Fig. 4a

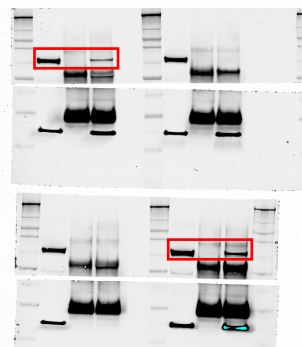

Fig. 4b

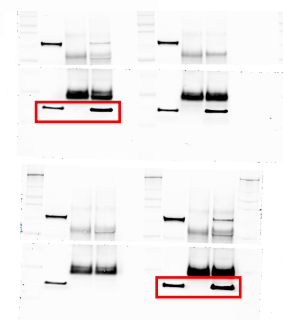

Fig. 4c

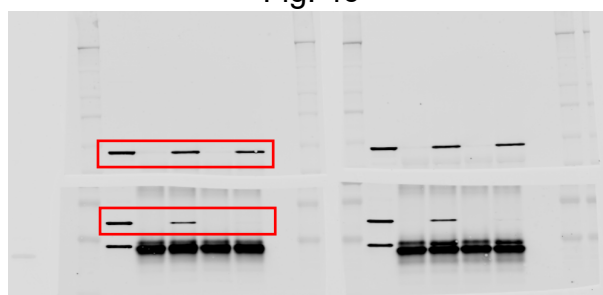

Fig. 4d

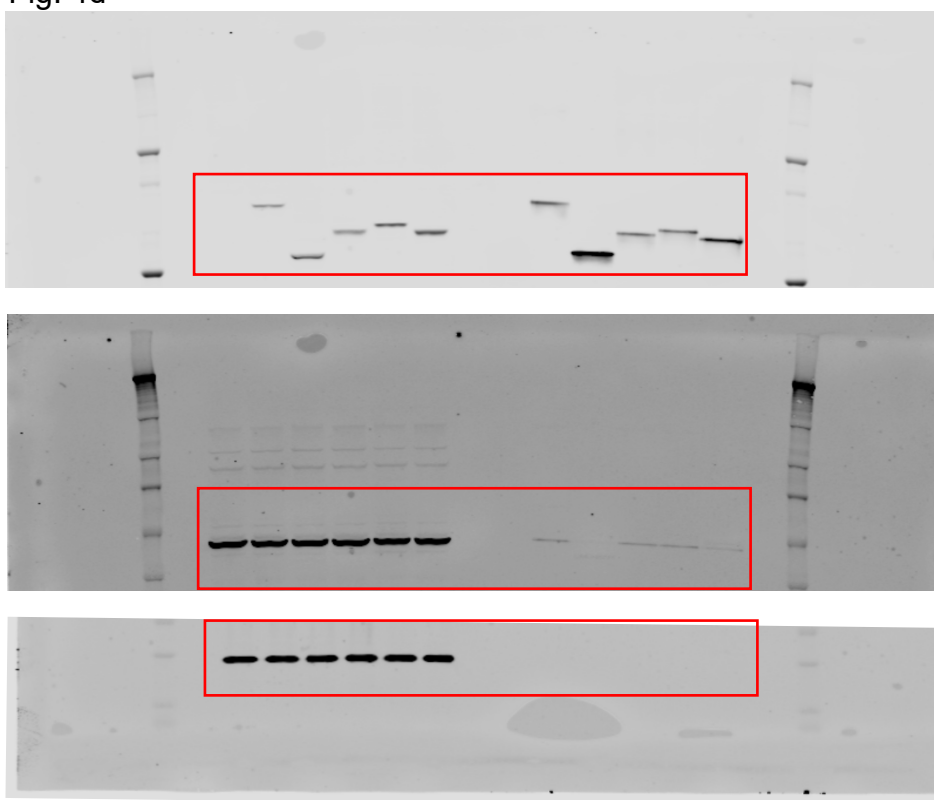

Supplement: Supplementary file 8 — Source data [file 41467_2023_40254_MOESM8_ESM.zip › Source data/Uncropped_gels_Fig3_4.pdf]

Fig. 5a

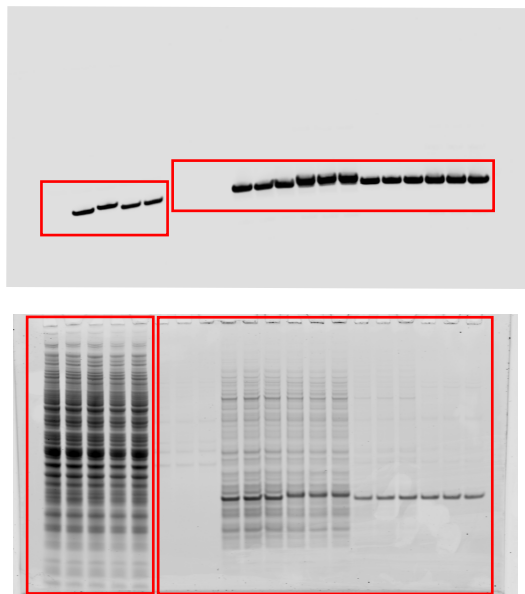

Fig. 7b

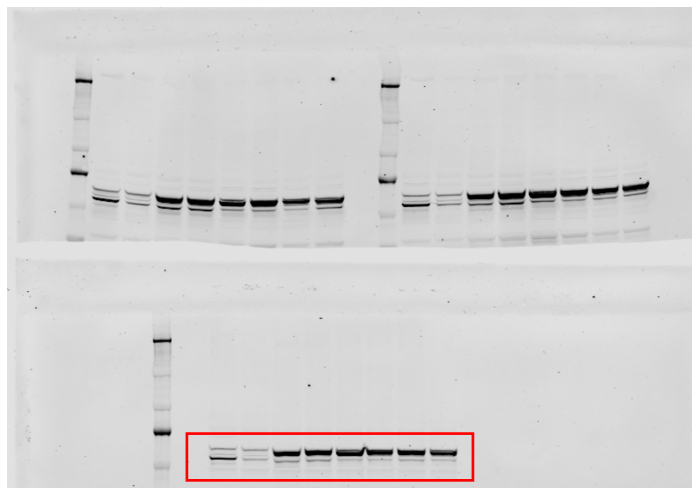

Fig. 7e

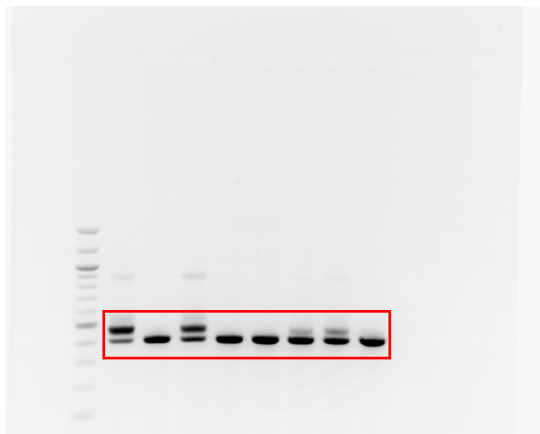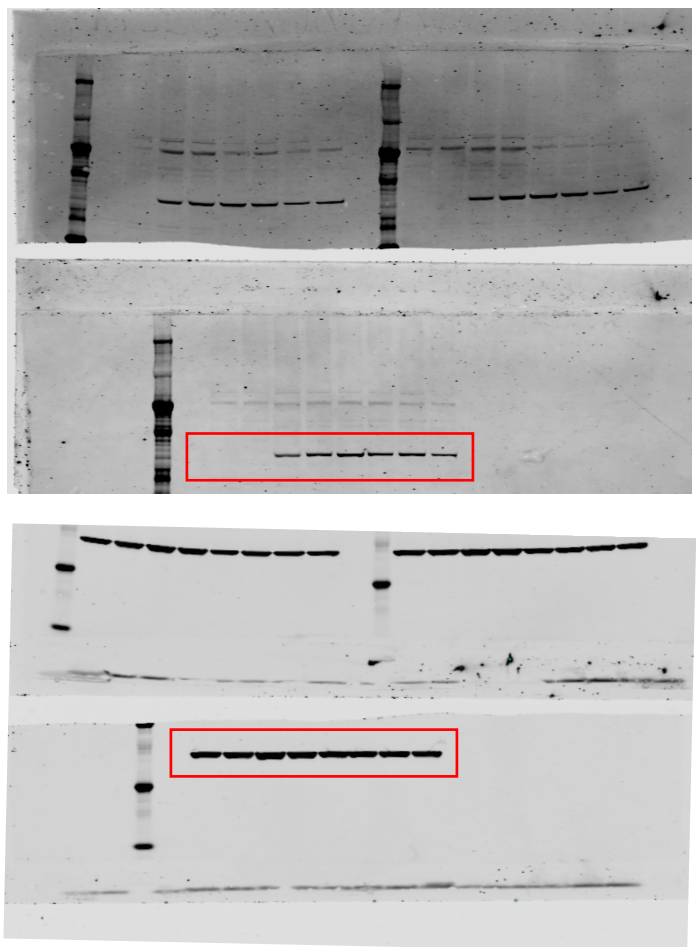

Supplement: Supplementary file 8 — Source data [file 41467_2023_40254_MOESM8_ESM.zip › Source data/Uncropped_gels_Fig5_7.pdf]

Fig. 8c

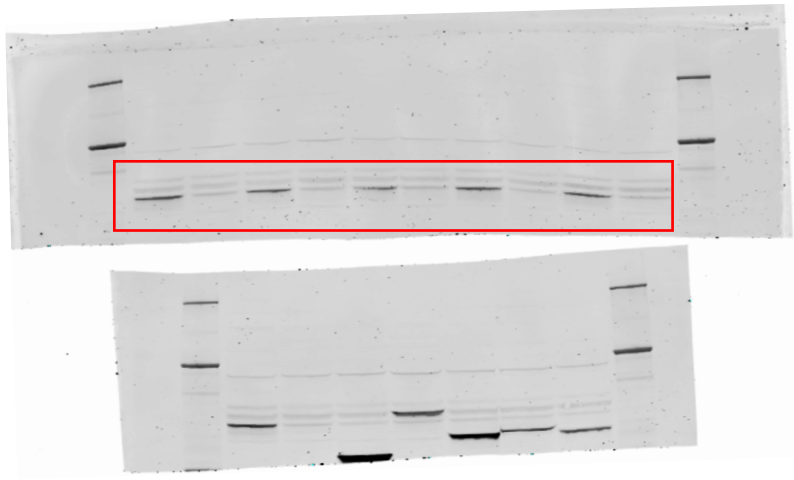

Fig. 8c

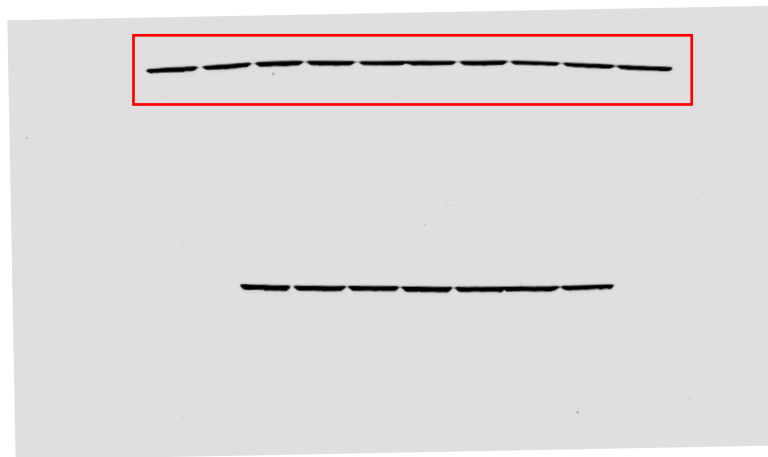

Fig. 8d

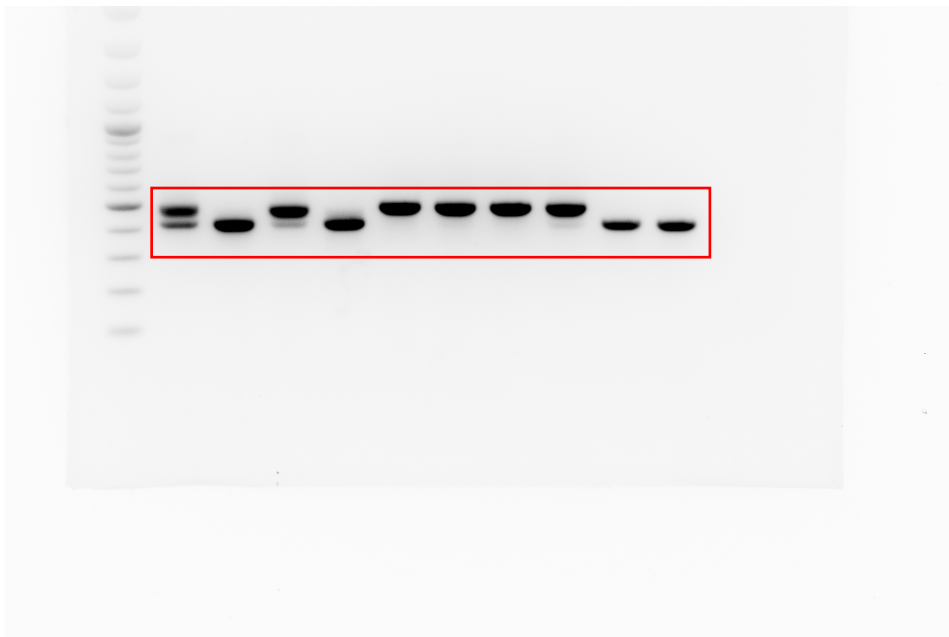

Supplement: Supplementary file 8 — Source data [file 41467_2023_40254_MOESM8_ESM.zip › Source data/Uncropped_gels_Fig8.pdf]

Supplementary Figure 10b-d

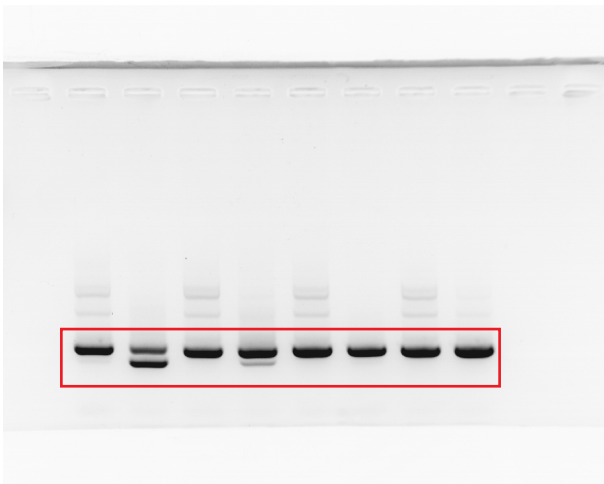

Supplementary Figure 10b-d

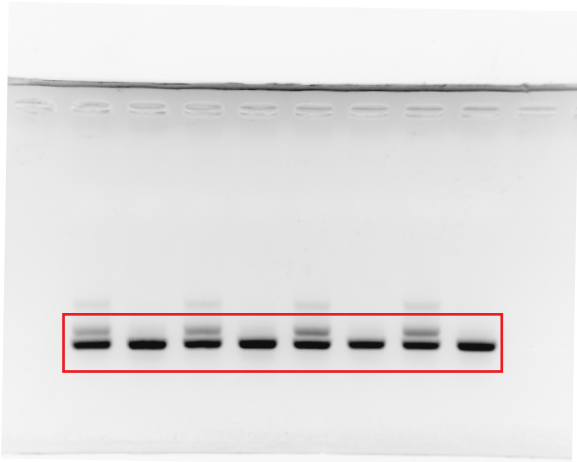

Supplement: Supplementary file 8 — Source data [file 41467_2023_40254_MOESM8_ESM.zip › Source data/Uncropped_gels_SuppFig10.pdf]
